# Synthetic bacteria for the detection and bioremediation of heavy metals

**DOI:** 10.3389/fbioe.2023.1178680

**Published:** 2023-04-13

**Authors:** Thi Duc Thai, Wonseop Lim, Dokyun Na

**Affiliations:** Department of Biomedical Engineering, Chung-Ang University, Seoul, Republic of Korea

**Keywords:** heavy metals, synthetic biology, bioremediation, whole-cell biosensor, genetically engineered microorganisms

## Abstract

Toxic heavy metal accumulation is one of anthropogenic environmental pollutions, which poses risks to human health and ecological systems. Conventional heavy metal remediation approaches rely on expensive chemical and physical processes leading to the formation and release of other toxic waste products. Instead, microbial bioremediation has gained interest as a promising and cost-effective alternative to conventional methods, but the genetic complexity of microorganisms and the lack of appropriate genetic engineering technologies have impeded the development of bioremediating microorganisms. Recently, the emerging synthetic biology opened a new avenue for microbial bioremediation research and development by addressing the challenges and providing novel tools for constructing bacteria with enhanced capabilities: rapid detection and degradation of heavy metals while enhanced tolerance to toxic heavy metals. Moreover, synthetic biology also offers new technologies to meet biosafety regulations since genetically modified microorganisms may disrupt natural ecosystems. In this review, we introduce the use of microorganisms developed based on synthetic biology technologies for the detection and detoxification of heavy metals. Additionally, this review explores the technical strategies developed to overcome the biosafety requirements associated with the use of genetically modified microorganisms.

## 1 Introduction

Heavy metals are elements with an atomic weight ranging from 63.5 to 200.6 g/mol and a density greater than 5 g/cm^3^ (Srivastava and Majumder, 2008). Certain heavy metals such as zinc (Zn), cobalt (Co), copper (Cu) and iron (Fe) are essential at a trace concentration to maintain life by involving in diverse biological functions (Appenroth, 2010; Kim et al., 2019a). Heavy metals accumulated at high concentrations can pose toxicity in living organisms by destroying membrane and DNA integrities and inhibiting the activity of proteins (Kim et al., 2015; Alrashed et al., 2021). For example, chromium (Cr), arsenic (As) and cadmium (Cd) induce DNA repair dysfunction and oxidative stresses, and thereby potentially cause cancers in human (Balali-Mood et al., 2021; Luo et al., 2021). Heavy metals inhibit enzymes by interfering with the formation of enzyme-substrate complexes, altering the functional integrity of active sites and compromising enzyme synthesis (Zackular et al., 2016; Lukowski and Dec, 2018). Interestingly, the continuous exposure to heavy metals such as As, Cd, Cu, lead (Pb), and Zn also disrupts the gut micro-ecosystem. The microbiome disruption may cause problems in immunity and resistance against bacterial infection, and eventually become vulnerable to diverse diseases (Choiniere and Wang, 2016; Zhai et al., 2019; Shao and Zhu, 2020).

For decades, human activities like farming, industrialization, and mining sharply facilitated the accumulation of heavy metals in the environment and thus threatened human wellness (Chandra et al., 2009; Nagajyoti et al., 2010; Bharagava et al., 2018) (Table 1). There have been many attempts to remediate contaminated environment, and significant attention has been paid to chemical and physical methods such as absorption, filtration, and chemical separations (Qasem et al., 2021). However, each method has its own disadvantages that limit its widespread use. For absorption, absorbents with different functional groups capturing heavy metals such as carbon, chitosan, or mineral-based absorbents are used (Qasem et al., 2021). The complex functionalization, instability, and low reusability of absorbents limit their use in industrial settings despite their high efficiency (Duan et al., 2020; Qasem et al., 2021). Membrane-based filtration is another method to separate heavy metals, which can be easily modulated by membrane pore size and permeability (Mukherjee et al., 2016). Although membrane-based filtration is easy to operate and highly efficient, frequent flux regeneration (cleaning) of membranes is necessary, which is incompatible with large-scale applications (Verma et al., 2021). Chemical methods use compounds to alter the valence state of heavy metals, such as the oxidation state, to diminish toxicity, or neutralize colloidal forces of heavy metals to precipitate and coagulate them. However, there are serious concerns about the chemical methods due to the utilization of toxic and health-harming agents to remove heavy metals; thus, chemical methods are considered non-sustainable (Bolisetty et al., 2019).

**TABLE 1:** Common heavy metals and their effects on health.

Heavy metal	Heavy metal sources	Impacts on health	References
Pb	Sewage sludge Gasoline Mining process	Systemic toxicity development in the neurological, cardiovascular, renal, hematological and reproductive systems IQ decrease in children	Matta and Gjyli (2016), Abdel-Razik et al. (2020)
As	Mine wastes Wood preservatives Fossil fuels Smelting and refining metals	Carcinogenicity Cardiovascular toxicity Pulmonary toxicity Neurotoxicity	Chung et al. (2014), Prakash and Verma (2021)
Hg	Cement production Metals production Combustion of fuels Gold mining	Impact on children development Injury to the central nervous system Toxicity in the brain and kidneys	Ha et al. (2017), Sundseth et al. (2017)
Cd	Combustion of fossil fuel Phosphate fertilizer Cement manufacture	Severe abdominal pain Diarrhea Potential prostate carcinogen Respiratory problems Renal dysfunction Disorders of calcium metabolism	Pan et al. (2010), Suhani et al. (2021)

To overcome the hurdles in the remediation by chemical and physical methods, bioremediation by microorganisms is gaining attention owing to their high efficiency and sustainability. To date, microorganisms isolated from contaminated area are commonly used for bioremediation because the organisms are tolerant against toxic chemicals and often possess the ability to convert toxic compounds into non-harmful ones. However, since natural microorganisms have not evolved to detoxify heavy metals but to survive in such harsh environments, they inevitably possess low bioremediation efficiency, still more efficient than the chemical and physical methods. Thus, there is a continuous demand for enhancing microbial detoxification capacity by the genetic reconstruction of their biochemical functions. One of the hurdles in constructing high-efficient bioremediation bacteria is how to engineer their biochemical ability to improve detoxification and tolerance, as well as how to engineer their intrinsic physiology for safe management.

Heavy metal biosensors are used to achieve *in situ* detection and efficient bioremediation in contaminated environments (Wei et al., 2014; Yin et al., 2016; Liu et al., 2022b). Biosensors utilize biological components such as enzymes, antibodies, nucleic acids, or whole cells to selectively detect particular heavy metals and convert the biorecognition event into measurable signals (fluorescent, optical, or electrical signal) (Velusamy et al., 2022). Among those biosensors, whole-cell biosensors can offer simplicity and low cost in *in situ* sensing, and also provide a chassis to embed remediation modules to eliminate heavy metals (Velusamy et al., 2022).

Synthetic biology applies engineering principles similar to those used in electronics to create and build synthetic organisms (Weiss et al., 2002), allowing for overcoming biotechnological challenges in genetic engineering, biochemical synthesis, and biological computation (Na et al., 2013; Chen et al., 2020b; Ren et al., 2020; Shaker et al., 2021). Recent advances in synthetic biology have enabled microorganisms to scavenge and bio-degrade a wide range of hazardous compounds, including aromatic compounds (Xiang et al., 2021), pesticides (Bhatt et al., 2021), microplastics (Miri et al., 2022), greenhouse gases (Tran et al., 2021), etc. In addition, synthetically engineered microorganisms with enhanced tolerance against toxic chemicals have also been developed for better bioremediation (Tran et al., 2021).

Our review focuses on recent advances in the microbial detection and bioremediation of heavy metals using the technologies and approaches of synthetic biology. In addition, the technical challenges and legal regulations associated with genetically modified microorganisms (GMMs) as well as the attempts to overcome the challenges are discussed.

## 2 Heavy metal resistance mechanisms

**FIGURE 1:**
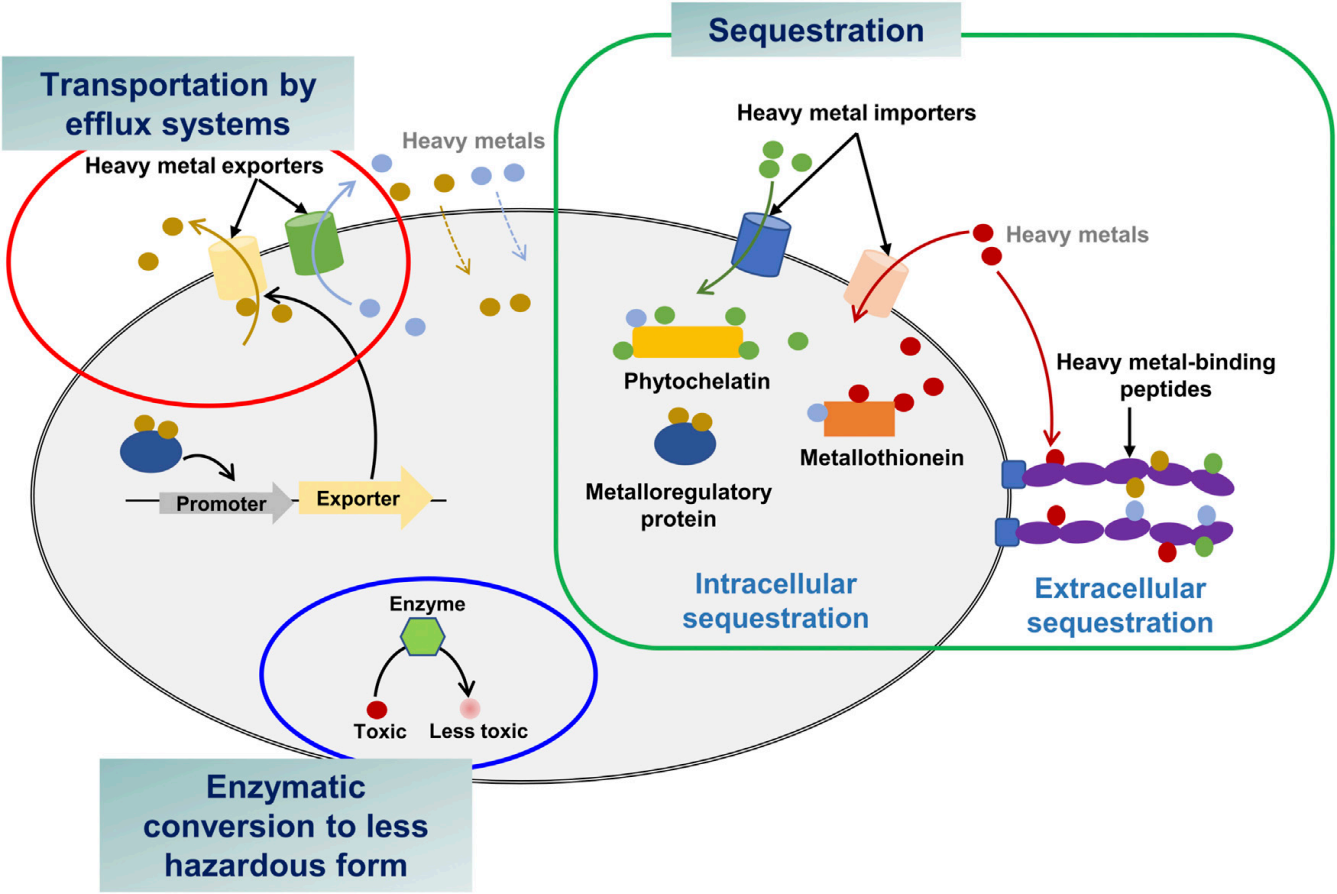
Heavy metal bioremediation mechanisms in bacteria.

Microorganisms in contaminated areas often develop tolerance against heavy metals and hence they could be suitable bacterial hosts to engineer (Malik, 2004). There are three different resistance mechanisms: transportation via efflux pump system, intra- and extracellular sequestration, and enzymatic conversion to a less toxic form (Figure 1) (Bruins et al., 2000). Considering these mechanisms, sequestration and enzymatic conversion are widely used strategies for application in bacteria to bioremediate heavy metals. In this section, the three mechanisms are introduced with examples of bacteria that are resistant to four significant toxic heavy metals (As, Hg, Cd, and Pb) (Balali-Mood et al., 2021) (Table 2).

**TABLE 2:** Natural heavy metal-resistant bacteria.

Bacteria	Heavy metal	Tolerance concentration	Types of environments	References
*Sporosarcina luteola* M10	As	0.2 M As^5+^	Electronic waste-contaminated soil	Salam et al. (2020)
		0.01 M As^3+^		
*Pseudomonas fluorescens*	Cd	0.002 M	Hussain Sagar lake	Rahman and Menon (2022)
*Stenotrophomonas* sp.	As	0.209 M As^3+^	Crven Dol mine	Bermanec et al. (2021)
		0.564 M As^5+^		
*Citrobacter farmeri* CFI-01	Pb	N.A	Phosphate mining wasteland	Li et al. (2022b)
*Pseudomonas aeruginosa*	Hg	0.07 M	Non-active sanitary landfill	Imron et al. (2019)
*Bacillus cereus* MG257494.1	Cd	0.01 M	Sediment samples from Al-Rahawy drain	El-Meihy et al. (2019)
*Alcaligenes faecalis* MG966440.1	Cd	0.01 M	Sediment samples from Al-Rahawy drain	El-Meihy et al. (2019)
*Rhizobium leguminosarum*	Cd	0.001 M	N.A	Lima et al. (2006)
*Klebsiella planticola*	Cd	0.015 M	Coastal salt marsh	Sharma et al. (2000)
*Pseudomonas aeruginosa*	Cd	0.005 M	Deep-sea hydrothermal vent	Wang et al. (2002)

N.A: not available.

### 2.1 Transportation by efflux systems

Efflux systems are found in diverse bacterial species, including *Pseudomonas aeruginosa*, *Candida albicans*, and *Escherichia coli* (Soto, 2013). They utilize efflux systems to export heavy metals from cytoplasm to extracellular regions in order to reduce toxicity (Bruins et al., 2000; Abdi et al., 2020). For example, As^3+^ is a toxic heavy metal form of As. Intracellular toxic As^3+^ is eradicated by the *ars* operon including *acr3* or *arsB* genes encoding As^3+^-efflux proteins (Yang et al., 2012). *Sporosarcina luteola* M10 isolated from contaminated soil of electronic wastes possesses the *arsB* gene for As transportation, allowing the bacterium to tolerate up to 0.2 M As^5+^ and 0.01 M As^3+^ (Salam et al., 2020). *Stenotrophomonas* sp. and *Microbacterium* spp. contain diverse As-efflux systems enabling a notably high As resistance (0.564 M As^5+^ and 0.209 M As^3+^) (Bermanec et al., 2021).

The *cad* operon is a well-known cluster of genes such as *cadABC* for Cd transportation (Smith and Novick, 1972). Numerous Cd-resistant bacteria have the *cadA* gene, including *Bacillus subtilis*, *Helicobacter pylori*, *P. putida*, and *Stenotrophomonas maltophilia* (Jebril et al., 2022). In *P. putida*, Cd^2+^-binding CadR, a transcription factor, promoted the expression of a CadA3 efflux protein as well as CzcCBA efflux proteins, contributing to Cd resistance (Liu et al., 2021). The CzcCBA efflux system ejects intracellular Cd^2+^ to the outer environment in cooperation with CadA3 efflux system. *P. fluorescens*, recently isolated from Hussain Sagar lake, exhibited Cd-tolerance up to 0.002 M Cd^2+^ (Rahman and Menon, 2022). Other bacterial species, such as *B. cereus* MG257494.1 and *Alcaligenes faecalis* MG966440.1 demonstrated high tolerance up to 0.01 M Cd^2+^ (El-Meihy et al., 2019).

Apart from the As- and Cd-efflux systems, bacteria also possess several other efflux systems to cope with the toxicity of other heavy metals. For instance, *Enterococcus hirae* harbors the *cop* operon, which contains the *copB* gene encoding a Cu-efflux pump to maintain Cu homeostasis (Solioz and Stoyanov, 2003). Interestingly, the *copB* gene also participates in the transport of silver (Ag) to the extracellular environment (Solioz and Odermatt, 1995). *E. coli* possesses the *zntA* gene that encodes an ATP-dependent efflux system, which confers resistance to Pb and Zn (Babai and Ron, 1998). Furthermore, *Cupriavidus metallidurans* CH34 (previously known as *Ralstonia metallidurans*) has the *cnrT* gene downstream of the *cnr* operon in plasmid pMOL28, which encodes a nickel-efflux system for nickel resistance (Nies, 2003).

### 2.2 Intra- and extracellular sequestration

Intracellular heavy metal ions can be sequestered by metal-capturing proteins, e.g., metallothioneins, glutathione (GSH), and metallochaperones (Bazzi et al., 2020). *Synechococcus* sp*.* has a metal resistance system containing the *smtA* gene encoding a Cd^2+^- and Zn^2+^-binding metallothionein (Bruins et al., 2000). In *Rhizobium leguminosarum*, GSH-mediated Cd sequestration through GSH-Cd chelation has been identified as a novel tolerance mechanism (Lima et al., 2006). A metallochaperone, PbrD protein, in *C. metallidurans* CH34 is able to sequester Pb within the cells and confer protection against toxic Pb (Taghavi et al., 2009).

Extracellular sequestration is to make metal ions insoluble and accumulates them outside bacteria. For instance, when sulfate-reducing bacteria produce vast amounts of hydrogen sulfide in the extracellular environment, sulfide precipitates metal cations (Igiri et al., 2018). Hydrogen sulfide produced from *Klebsiella planticola* under anaerobic conditions and that from *P. aeruginosa* under aerobic conditions precipitates Cd^2+^ as CdS (Sharma et al., 2000; Wang et al., 2002). *Citrobacter farmeri* CFI-01, isolated from a phosphate mining wasteland, can produce soluble phosphate that precipitates Pb^2+^ (Li et al., 2022b).

### 2.3 Conversion of heavy metals into less hazardous forms

Several microorganisms have evolved mechanisms to reduce the sensitivity of cellular components to heavy metals by converting a toxic heavy metal into a less toxic form. Due to the affinity of mercury (Hg) for thiol groups, proteins with a thiol group can be inactivated by Hg. Hg resistance is a typical example of enzymatic detoxification (Misra, 1992). The *mer* operon of Hg-resistant bacteria such as *Shigella flexneri*, *Staphylococcus aureus*, *P. stutzeri*, *P. aeruginosa*, *K. pneumoniae*, *Mycobacterium marinum*, and *Enterobacter* contains the *merA* (mercuric ion reductase) and *merB* genes (organomercurial lyase). The lyase demethylates highly neurotoxic methylated mercury (MeHg) and releases toxic Hg^2+^, and then Hg^2+^ is converted to a volatile and nontoxic Hg^0^ by the reductase (Bruins et al., 2000; Dash and Das, 2012). The operon also contains the *merT* and *merP* genes encoding the proteins responsible for the transportation of Hg^2+^ from cytoplasm to outside. The cooperative Hg detoxification system effectively protects the host (Bruins et al., 2000).

Cr exists in one of two stable oxidation states in the environment, Cr^6+^ and Cr^3+^, and Cr^3+^ is a less hazardous form of Cr^6+^ (Mitra et al., 2022). Microorganisms can enzymatically reduce Cr^6+^ to Cr^3+^ for tolerance. Under aerobic conditions, the reduction is mediated by soluble and membrane-bound reductases such as ChrR and YieF. ChrR catalyzes the reduction of Cr^6+^ to Cr^5+^, and then to Cr^3+^. On the other hand, the YieF enzyme can directly convert Cr^6+^ into Cr^3+^ without the intermediate formation of Cr^5+^. Under anaerobic conditions, anaerobic bacteria such as *P. dechromaticanse* and *Enterobacter cloacae* use membrane-bound reductases such as flavin reductases, cytochromes, and hydrogenases to carry out the reduction of Cr^6+^ by providing electrons from hydrogen, carbohydrates, NAD(P)H, and endogenous electrons (Lovley and Phillips, 1994; Thatoi et al., 2014).

## 3 Heavy metal detection

When constructing synthetic bacteria for bioremediation, the first step is to implement a heavy metal detection system for the transition from a resting state to a bioremediating state. The synthetic bacteria sensing heavy metals can also be used as a whole-cell biosensor, which can provide several benefits to overcome industrial limitations: rapid and *in situ* detection, eco-friendliness, and cost-effectiveness (Gupta et al., 2019; Inda and Lu, 2020).

Whole-cell biosensors harbor genetic elements/circuits that recognize a target heavy metal. Natural bacteria isolated from heavy metal-polluted areas are the source of new genetic elements for biological sensor machineries (Valls and de Lorenzo, 2002). Heavy metal-responsive transcription factors and two-component regulatory systems (TCRS) are the key components of whole-cell biosensors for heavy metal detection. Heavy metal-responsive transcription factors are the proteins that regulate the expression of target genes via heavy metals binding and TCRS consists of a sensor kinase and a response regulator. The sensor kinase senses environmental signals such as heavy metals, and the kinase in turn, activates the response regulator via phosphorylation. The phosphorylated regulator initiates the expression of target genes required for detoxification (Wang and Buck, 2012). For whole-cell biosensors, the transcription factors or response regulators are engineered to transcribe a fluorescent or bioluminescent protein in response to the presence of heavy metals in the environment.

### 3.1 Metal-responsive transcription factor-based biosensors

**FIGURE 2:**
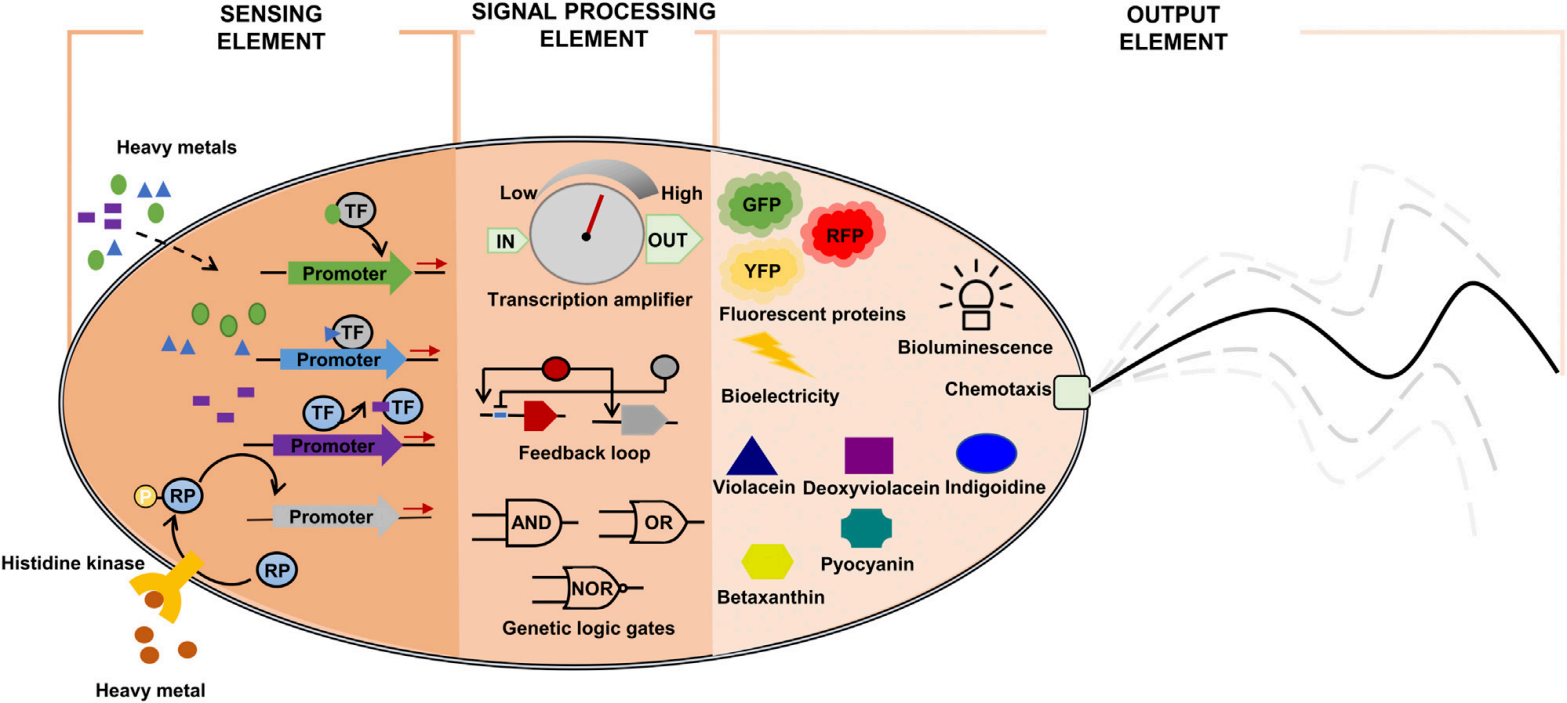
Components of synthetic bioremediation bacteria for heavy metal detection. GFP: green fluorescent protein; RFP: red fluorescent protein; RP: regulatory protein; TF: heavy metal-responsive transcription factor; YFP: yellow fluorescent protein.

A simple genetic detection circuit can consist of sensor and transducer elements (Bereza-Malcolm et al., 2015). The sensor element is generally a transcription factor perceiving the presence of heavy metal. The signal-processing element transforms the signal from the sensor element into a detectable optical (fluorescence or pigment compounds) or electrical signal for real-time analysis (Figure 2). The signal-processing element often incorporates an amplifier, feedback loop, and/or logic gates to tune the output/input signal ratio (Figure 2) (Liu et al., 2022a).

The sensor element can be implemented using transcription factors that are responsive to heavy metals (Osman and Cavet, 2010). Once activated, the transcription factors initiate the production of detectable signal proteins (color, fluorescence, or electrical signals). Thus, the focus in whole-cell biosensor engineering is to implement a module of transcription factor-signal emitting protein, which can accurately transduce input signal levels (heavy metal concentrations) to proportionally output signal levels (optical or electrical signals) (Table 3).

**TABLE 3:** Synthetic biology approaches applied to whole-cell biosensors for heavy metal detection.

Heavy metal	Engineered bacteria	Engineering approach	Signal-processing element	Limit of detection	Output element	Application	References
As	*E. coli* DH5α	The mutation of the −10 site and the addition of an extra ArsR-binding downstream of the −10 site were performed in an arsenite-controlled P_*ars*_ promoter	N.A	10 nM	Green fluorescent protein	River water	Chen et al. (2019)
As	*E. coli* DH5α	The ArsR-binding site was placed to overlap the core region (between −35 and −10 site) of the P_*ars*_ promoter. The ArsR expression level was driven by P_*lacV*_ promoter	N.A	1.85 nM	Green fluorescent protein LacZ (β-galactosidase)	Groundwater	Chen et al. (2022)
As	*E. coli* DH5α	P_*ars*_ promoter controlled the expression of ArsR regulator and mutant LuxR	P_*luxI*_ promoter controlled the expression of mCherry protein and mutant LuxR as the positive feedback	0.1 µM	mCherry protein	N.A	Jia et al. (2019)
As	*E. coli* DH5α	P_*nikA*_ promoter of *nikABCDE* operon was used to control the expression of *egfp* gene	N.A	N.A	Green fluorescent protein	Soil sample	Yoon et al. (2016)
Hg	*P. aeruginosa* PAO1	P_*mer*_ promoter was used to control the expression of pyocyanin synthesis genes (*phzM*, *phzS*) and *merR* gene encoding MerR regulator	N.A	10 nM	Pyocyanin (Blue-green pigment)	Lake water	Wang et al. (2020)
Hg/Cd	*E. coli* TOP10	P_*mer*_ promoter controlled the *merR* and *egfp* gene expression. P_*cad*_ promoter controlled the *cadR* and *mcherry* gene expression	N.A	0.0098 µM Hg^2+^ 0.098 µM Cd^2+^	Green fluorescent protein (Hg) mCherry protein (Cd)	N.A	Hui et al. (2022b)
Hg	*E. coli* TOP10	P_*mer*_ promoter controlled *merR* gene and *C. violaceum*-derived *vioABCDE* operon	N.A	Lag phase: 0.006 μM Exponential phase: 0.39 μM	Violacein (Navy pigment)	Environmental water	Guo et al. (2021a)
Hg	*E. coli* MG1655	P_*mer*_ promoter controlled the expression of *merR* gene and *luxCDABE* operon. nMagHigh and pMagHigh proteins were continuously expressed on the outer membrane via fusion with OmpX protein	N.A	1.25 nM	Bioluminescence (Luciferase)	Lake water	Chen et al. (2020a)
Cd	*P. putida* KT2440	P_*cad*_ promoter controlled the expression of mCherry protein. CadR regulator was expressed by P_*lteto1*_	TetR and P_*lteto1*_ promoter were used as the negative feedback amplifier	0.1 nM	mCherry protein	River water	Zhang et al. (2021)
Cd	*E. coli* TOP10	P_*cad*_ promoter controlled the expression of *cadR* gene, *Streptomyces lavendulae*-derived *bpsA* gene and *P. aeruginosa* PAO1-derived *pcpS* gene	N.A	0.049 µM	Indigoidine (Blue pigment)	Environmental water	Hui et al. (2022a)
Pb	*E. coli* DH5α	P_*pbr*_ promoter controlled the expression of *egfp* gene. P558 promoter constitutively expressed PbrR regulator	Uncoupled feedback circuit	50 nM	Green fluorescent protein	Milk and sewage sample	Du et al. (2019)
Pb	*E. coli* TOP10	P_*pbr*_ promoter controlled the expression of *pbrR* gene and *C. violaceum*-derived *vioABCE* genes	N.A	2.93 nM	Deoxyviolacein (Blue pigment)	Tap water Lake water Soil extract	Hui et al. (2022c)
Cu	*E. coli* TOP10	P_*cusC*_ regulated by CusSR system was used to express *gfp* gene	N.A	12 µM	Green fluorescent protein	N.A	Wang et al. (2013)
Cu	*E. coli* XL1-Blue	P_*cusC*_ promoter regulated by CusSR system was used to express *cusR* with the *gfp* gene	Additional expression CusR regulator encoded by *cusR* gene was used as the positive feedback loop	4 µM	Green fluorescent protein	N.A	Ravikumar et al. (2012)
Cu	*C. metallidurans*	P_*copQ*_ regulated by CopSR system was used to express the betaxanthin synthesis gene (*dod*) from *Mirabilis jalapa* plant	N.A	87.3 μM	Betaxanthin (Yellow pigment)	N.A	Chen et al. (2017)
Cu	*E. coli* Rosetta	P_*cusC*_ promoter regulated by CusSR system was used to express riboflavin synthesis gene (*ribB*). T7 promoter expressed *oprF* gene encoding porin to export riboflavin to outer space	N.A	28.5 µM	Voltage mediated by the extracellular electron transfer with the aid of extracellular riboflavin	Wastewater	Zhou et al. (2021)
Zn	*E. coli* XL1-Blue	P_*zraP*_ promoter regulated by ZraSR system expressed *gfp* gene	N.A	1 µM	Green fluorescent protein	N.A	Ravikumar et al. (2011)
Zn	*E. coli* XL1-Blue	P_*zraP*_ promoter regulated by ZraSR system expressed *hydG* (*zraR*) and *gfp* gene	A positive feedback loop was built by expressing HydG (ZraR) protein	10 µM	Green fluorescent protein	N.A	Pham et al. (2013)
U	*C. crescentus*	UzcRS and UrpRS system were used to regulate the expression of tripartite green fluorescent protein	AND gate circuit was built based on the combination of different green fluorescent protein fragments, whose expressions were separately controlled by UzcRS and UrpRS system, to become the functional green fluorescent protein	1 µM	Tripartite green fluorescent protein	Groundwater	Park and Taffet (2019)
Cd	*E. coli* RP437	A computationally redesigned ribose binding protein sensed Cd	N.A	50 µM	Chemotaxis	N.A	Li et al. (2022a)
Tb	*E. coli*	Fe^3+^-binding loop was replaced by a Tb^3+^-binding peptide sequence in histidine kinase (PmrB). P_*mrC*_ promoter regulated by PmrB/PmrA system expressed *gfp* gene or *cheZ* gene	N.A	1.0 μM	Green fluorescent protein Chemotaxis	N.A	Liang et al. (2013)

N.A: not available.

#### 3.1.1 As biosensor

Seven family members of metal-binding transcription factors have been identified in bacteria: ArsR-SmtB, NikR, DtxR, MerR, Fur, CopY, and CsoR-RcnR (Osman and Cavet, 2010). Each family takes part in sensing a distinct group of metals. ArsR is a repressor protein belonging to the ArsR-SmtB family. Upon binding to As, ArsR is detached from its cognitive promoter and thereby initiates the transcription of the *ars* operon involved in As detoxification (Busenlehner et al., 2003; Osman and Cavet, 2010).

**FIGURE 3:**
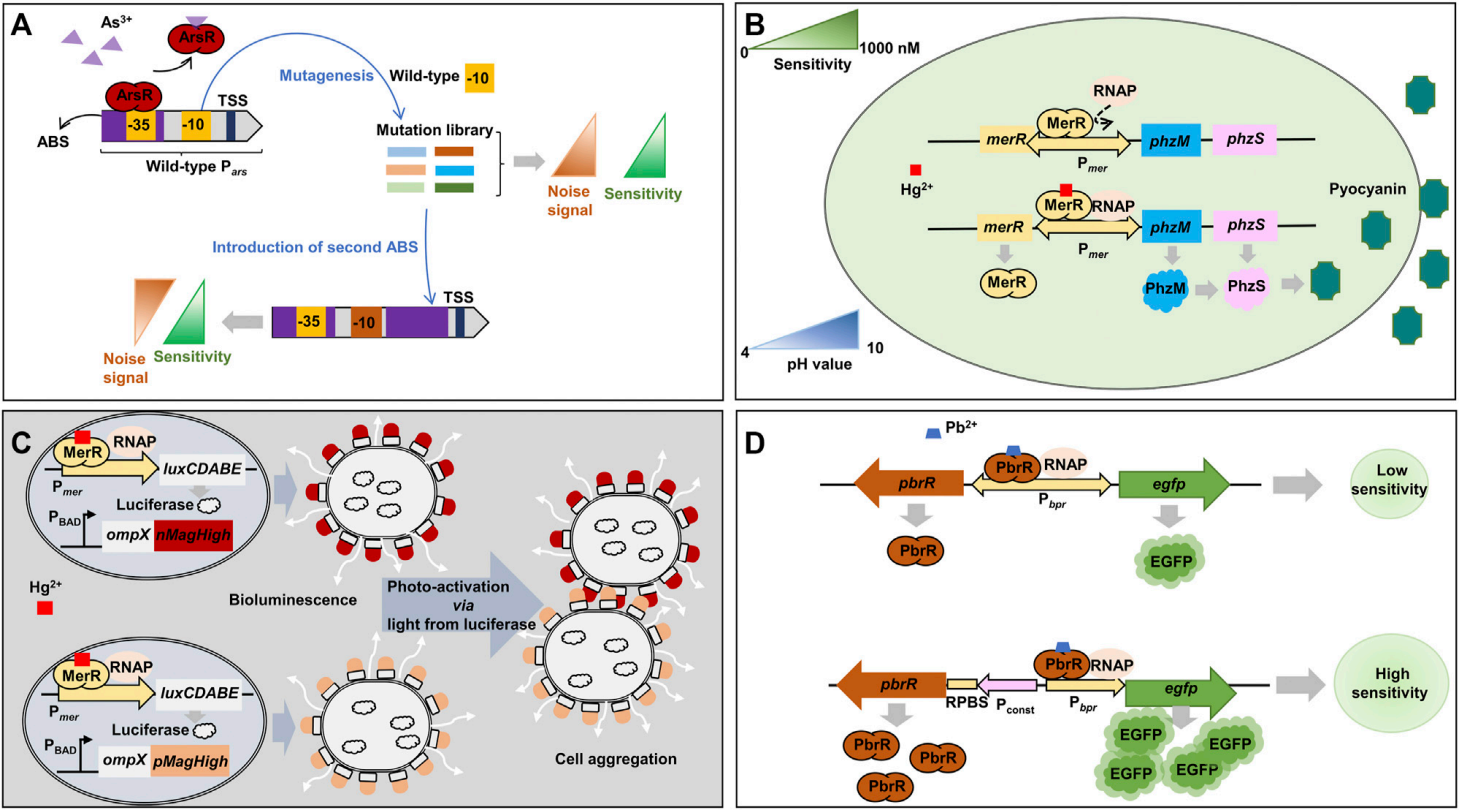
Representative synthetic biology approaches for metal-responsive transcription factor-based whole-cell biosensors. **(A)** Promoter engineering by screening −10 site mutations and addition of an extra ArsR-binding site (Chen et al., 2019). **(B)** Use of pyocyanin, a blue-green pigment, as an optical output signal by swapping the structural genes of *mer* operon with pyocyanin synthesis genes (Wang et al., 2020). **(C)** Enhancement of bioluminescent signal by biofilm formation via the cell aggregation by photoswitchable proteins, nMagHigh and pMagHigh, expressed on the outer membrane (Chen et al., 2020a). **(D)** Optimization of heavy metal-responsive transcription factor expression to increase biosensor sensitivity (Du et al., 2019). *luxCDABE*: luciferase; *nMagHigh*: photoswitchable protein nMagHigh; *ompX*: outer membrane protein X; *pMagHigh*: photoswitchable protein pMagHigh; ABS: ArsR-binding site; RNAP: RNA polymerase; ArsR: arsenic-sensing transcriptional regulator; EGFP: enhanced green fluorescent protein; MerR: mercury-sensing regulator; P_const_: moderate constitutive P558 promoter; P_*ars*_: arsenic-inducible promoter; P_BAD_: *ara*BAD operon promoter; PbrR: lead-sensing transcriptional regulator; PhzM: phenazine-specific methyltransferase; PhzS: flavin-containing monooxygenase; P_*mer*_: mercury-inducible promoter; P_*pbr*_: lead-inducible promoter; RPBS: regulator protein-binding site; TSS: transcription start site.

Chen et al. engineered the ArsR-responsive P_*ars*_ promoter and optimized ArsR expression level to improve the limit of detection (LOD) and decrease the background signal of the biosensor based on ArsR (Figure 3A) (Chen et al., 2019). A library of mutated −10 sites of P_*ars*_ promoter was generated in order to find mutant promoters showing higher sensitivity towards As as well as lower background signal than the wild-type P_*ars*_ promoter. One P_*ars*_ promoter variant (P_*ars*D_) containing a mutation within −10 site (GACACT) showed a medium-high fluorescent signal in response to 1 μM of As^3+^. In addition to the promoter engineering, the location of ArsR-binding site was further optimized to reduce background noise signal. When the second ArsR-binding site was placed downstream of the −10 site of the promoter for tight repression, the background signal was remarkably decreased from 2352 to 142, thus improving the induction ratio (fluorescence signal/background noise) from 16.8 to 179.3. Consequently, the engineered P_*ars*_ promoter was able to detect As as low as 10 nM. In a following study, the ArsR-binding site was placed to overlap the core region (between −35 and −10 site) of the P_*ars*_ promoter after the alignment of ArsR-binding site sequence with a library of RNA polymerase binding sites (−35 and −10 sites) and −35 site restructure in ArsR-binding site sequence (Chen et al., 2022). In addition, the ArsR expression level was also optimized. Thus, the induction ratio of the newly designed promoter was further improved up to 183.52. The implemented whole-cell biosensor could detect As concentration as low as 1.85 nM and allowed on-the-spot As identification.

Jia et al. incorporated a LuxR-based positive feedback into ArsR-based As biosensor to amplify output signal as well as to improve sensitivity (Jia et al., 2019). The LuxR protein lacked its N-terminal region (Δ2-162) and thus showed a constitutive transcription initiation activity even in the absence of its activator (Nistala et al., 2010). Two copies of the mutant *luxR* gene were incorporated into the biosensor. The first copy was under the control of P_*ars*_ and the second copy was placed downstream of the *mcherry* gene in a bicistronic configuration regulated by P_*luxI*_. Thus, once LuxR is produced by the activation of P_*ars*_ promoter in the presence of As, the LuxR triggers the positive feedback by transcribing mCherry and *luxR* genes via P_*luxI*_. The whole-biosensor showed a time-dependent response to As due to the positive feedback and could detect As concentration as low as 0.1 µM which was lower than the safety concentration of As in drinking water recommended by the World Health Organization.

*E. coli* harbors the nickel (Ni) ABC-type transporter, NikABCDE, and this operon is transcriptionally repressed by Ni via the NikR repressor (Leitch et al., 2007). Interestingly, it has been discovered that As impeded the repression activity of NikR protein and thereby As activated the transcription of the *nik* operon (Yoon et al., 2016). This discovery exemplifies an unpredicted interaction between heavy metals and transcription factors, which requires a deep understanding of the regulation mechanisms when constructing whole-cell biosensors.

#### 3.1.2 Hg and Cd biosensor

The MerR transcription factor family senses several different heavy metals, for example, MerR for Hg, PbrR for Pb, CadR for Cd, CueR for Cu, ZntR for Zn, and CoaR for Co (Brown et al., 2003). *P. aeruginosa* PAO1 has been engineered to implement an optical biosensor for Hg detection (Figure 3B) (Wang et al., 2020). Specifically, the endogenous *mer* operon under the control of a Hg-responsive regulator (MerR) was engineered to transcribe a phenazine-specific methyltransferase (*phzM*) and a flavin-containing monooxygenase (*phzS*) to synthesize pyocyanin when the microorganism was exposed to Hg. Pyocyanin is a soluble blue-green pigment (optical color signal). The engineered biosensor operated stably at a wide range of pH from 4 to 10 and showed an LOD of 10 nM Hg^2+^.

A colorimetric biosensor for Hg detection was constructed by employing the *vioABCDE* operon derived from *Chromobacterium violaceum,* which produces violacein, a navy pigment (Guo et al., 2021a). The P_*mer*_ promoter and the Hg-responsive transcription factor-encoding *merR* gene obtained from the transposon Tn21 in *E. coli* were utilized to regulate the expression of the *vioABCDE* in response to Hg. Navy color could be detectable after 1 hour of induction with 8 μM Hg^2+^. The whole-cell biosensor in a lag phase exhibited low LOD (0.006 μM) but a narrow detection range (0–0.012 μM), whereas that in an exponential phase displayed a wider detection range (0.78–12.5 μM) but higher LOD (0.39 μM).

A dual-output signal biosensor has been developed to simultaneously detect Hg and Cd using MerR and CadR transcriptional factor (Hui et al., 2022b). The MerR was used to regulate the expression of green fluorescent protein (GFP) (green color) in response to Hg and CadR for mCherry protein (red color) in response to Cd. The sensor could quantitatively assess the concentrations of Hg and Cd within the range of 0–5 μM and 0–200 μM, respectively.

To improve the detection range towards Hg, *E. coli* MG1655 has been engineered based on bioluminescence (light) and light-induced artificial biofilm (Figure 3C) (Chen et al., 2020a). For the detection of Hg at very low concentration, *merR* gene was cloned under the control of P_*mer*_ promoter as a positive feedback loop. Once MerR is activated by Hg, the sensor continuously produces MerR as well as other downstream genes, *luxCDABE*, for bioluminescence. To strengthen the signal, the sensor employed nMagHigh and pMagHigh proteins, which were displayed on the cell’s surface with the aid of the circularly permutated outer membrane protein, OmpX. The two proteins were photoswitchable proteins, and once activated by light emitted from luciferase reporter protein encoded by *luxCDABE,* they interacted with each other and aggregated bacterial cells. The cell aggregation strengthened the bioluminescence signal. Consequently, the biosensor could detect Hg in the range of 1.25 nM and up to 1,000 nM Hg^2+^ (Chen et al., 2020a).

*P. putida* KT2440 has been engineered to improve sensitivity and specificity to Cd (Zhang et al., 2021). In the whole-cell biosensor, CadR expression was maintained at a low level by a negative feedback loop and the *mcherry* gene was regulated by CadR. Due to the low level of CadR, the sensor showed a very low background signal leading to greater sensitivity and better specificity.

Indigoidine, a blue pigment, has been utilized as an output signal in a Cd-sensing whole-cell biosensor (Hui et al., 2022a). The Cd-sensing system was composed of P_*cad*_ promoter and *cadR* gene originated from *P. putida*. Upon activation of CadR by Cd, P_*cad*_ promoter initiated the expression of the *bpsA* gene (*Streptomyces lavendulae*) and the *pcpS* gene (*P. aeruginosa* PAO1) for indigoidine biosynthesis. The blue color of the indigoidine could be discerned with naked eyes after 30 min of induction with 100 µM of Cd^2+^. Notably, this pigment-based biosensor showed no detectable background signal in contrast to the previously used fluorescence-based biosensor for Cd detection. The LOD of this indigoidine-based biosensor was 0.049 µM of Cd^2+^, and it was successfully applied to verify the presence of Cd in environmental water samples.

#### 3.1.3 Pb biosensor

The Pb-sensing protein, PbrR, has a high affinity to Pb^2+^, but it also interacts with other divalent metal ions such as Zn^2+^, Cu^2+^, and Cd^2+^. Thus, PbrR has been engineered via mutagenesis for higher specificity to Pb^2+^ than other divalent metal ions (Jia et al., 2020b). The specificity was measured by the growth of the engineered bacterial cells by implementing the cell to produce ampicillin-resistant enzymes by the mutant PbrR under diverse metal ions. Of many mutants, a mutant (D64A/L68S) was identified to improve specificity: the affinity (growth rate) to Pb^2+^ of the mutant was two-fold higher than wild-type, while the affinity to other metal ions (Zn, Cu, and Cd) was comparable with wild-type.

An *E. coli* whole-cell biosensor for Pb detection was constructed with the PbrR-responsive promoter (P_*pbr*_) and downstream genes of *pbrR* and *egfp* to keep PbrR at an optimal level (Figure 3D) (Du et al., 2019). In a previous study on a biosensor for Cd, the low-level expression of the transcription regulator increased sensitivity and specificity by reducing background noise (Zhang et al., 2021). However, the Pb biosensor showed an increased background signal even though the PbrR was at a low level. It could be because that the level of PbrR was too low to efficiently repress the promoter activity resulting in leaky expression of the *egfp* gene. When the *pbrR* gene was expressed by a moderate constitutive promoter and the *egfp* gene was still under the control of P_*pbr*_ promoter, the sensor module showed higher sensitivity within a range of 50 nM - 10 μM Pb^2+^. This result represents the importance of expression optimization for the reliable operation of biosensors.

Hui et al. constructed four variants of the violacein gene cluster of *vioABCDE* (*C. violaceum*) to develop multiple-pigment biosensors for detecting Pb (Hui et al., 2022c): *vioABE* genes for prodeoxyviolacein (green), *vioABDE* genes for proviolacein (blue), *vioABCE* genes for deoxyviolacein (purple), and *vioABCDE* genes for violacein (navy). For the expression of the cluster variants, the P_*pbr*_ promoter and *pbrR* gene obtained from *C. metallidurans* was utilized. The biosensors successfully produced the respective pigments upon detection of Pb. Of the biosensors, the deoxyviolacein biosensor exhibited the widest detection range (2.93–6,000 nM) and the lowest LOD (2.93 nM) among the other pigment biosensors.

### 3.2 Two-component regulatory system-based biosensors

Bacteria possess another machinery, called TCRS, to sense extracellular environments, such as chemical and physical changes (Bijlsma and Groisman, 2003; Casino et al., 2010). This system comprises two main components, a histidine kinase and a regulator protein. The kinase contains a periplasm-located loop formed by two transmembrane segments for receiving signals and a cytoplasm-positioned transmitter domain found within the final transmembrane segment for controlling a downstream regulator via phosphorylation or dephosphorylation. When the regulator is phosphorylated, it becomes a transcription activator and initiates the transcription of downstream genes to respond to environmental changes (Casino et al., 2010).

TCRS offers several advantages for developing whole-cell biosensors over the transcription factor-based approach. TCRS can sense extracellular as well as intracellular stimuli, but transcription factors can sense only the intracellular changes. Thus, transcription factor-based biosensors require additional modules to transmit the input signal (e.g., extracellular heavy metals) into the cytoplasm (Ulrich et al., 2005; Mimee et al., 2018). In addition, the output signals of TCRS are relatively less impacted by the levels of histidine kinase and regulator, which exempts us from optimizing the expression levels of individual biosensor component genes (Batchelor and Goulian, 2003; Shinar et al., 2007). Thus, TCRS responses are more secured from the internal noises generated in the gene expression of component genes.

#### 3.2.1 Cu biosensor

Cu TCRS is composed of CusS for sensing Cu and CusR for regulating P_*cusC*_ promoter, and this system has been widely studied and utilized to construct Cu whole-cell biosensors (Rademacher and Masepohl, 2012; Yu et al., 2022). The native CusSR TCRS of *E. coli* MG1655 was used to produce green fluorescence by expressing the *gfp* gene under Cu-responsive P_*cusC*_ (Wang et al., 2013). The LOD of the system was as low as 12 µM of Cu^2+^. Ravikumar et al. developed a positive feedback loop by co-expressing *cusR* with the *gfp* gene via P_*cusC*_ promoter to enhance Cu sensitivity (Ravikumar et al., 2012). The LOD of the biosensor was improved by 10-fold (4 μM) compared with the native system.

Chen et al. improved the specificity of a Cu biosensor based on the CopSR regulatory system, another Cu TCRS discovered in *C. metallidurans*. To find a promoter specific to Cu, they screened *cop* promoters (P_*copA*_, P_*copH*_, P_*copT*_, P_*copM*_, P_*copF*_, P_*copL*_, P_*copQ*_, and P_*copE*_) regulated by CopR in the presence of 1 mM of Cu^2+^, Fe^3+^, Co^2+^, Ni^2+^, Zn^2+^, or Pb^2+^ (Chen et al., 2017). These promoters were designed to drive the expression of the *rfp* gene encoding red fluorescent protein, thereby facilitating the quantitative assessment of individual promoter activity in terms of protein expression. Of the eight promoters, P_*copQ*_ showed high Cu^2+^-specific induction pattern and thus was employed in the Cu biosensor. For color detection, the Cu^2+^-specific P_*copQ*_ controlled the expression of the *Mirabilis jalapa*-derived *dod* gene (4,5-dihydroxy-phenylalanine dioxygenase), which is involved in the conversion of L-dihydroxy-phenylalanine (L-DOPA) to betaxanthin, a yellow pigment. The addition of L-DOPA in media facilitated the production of betaxanthin as a reporter.

**FIGURE 4:**
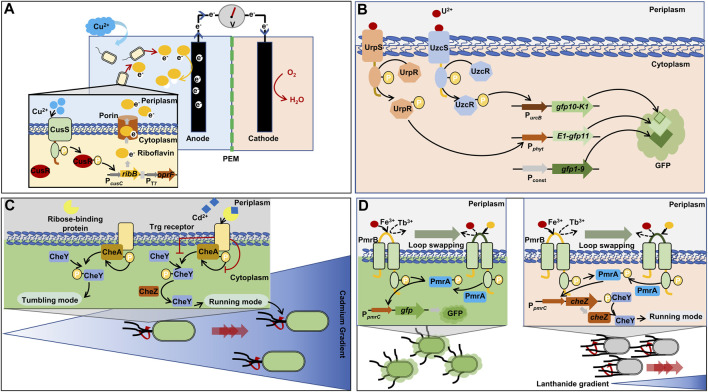
Representative synthetic biology approaches for two-component regulatory system (TCRS)-based whole-cell biosensors. **(A)** Rapid and inexpensive electrical detection of cooper by riboflavin as an output signal (Zhou et al., 2021). **(B)** Combination of two TCRS systems for the specific detection of uranium and tripartite green fluorescent protein cooperation (Park and Taffet, 2019). **(C)** Chemotaxis towards cadmium via retailored ribose binding protein for cadmium binding (Li et al., 2022a). **(D)** Formation of a lanthanide-sensing component by exchanging iron-binding motif of histidine kinase with lanthanide-binding peptide (Liang et al., 2013). *cheZ*: phosphatase; *gfp*: green fluorescent protein; *gfp10*: β-strand 10 of green fluorescent protein; *gfp11*: β-strand 11 of green fluorescent protein; *gfp1-9*: β-strand from 1 to 9 of green fluorescent protein; *oprF*: outer membrane porin F; *ribB*: 3,4-dihydroxy-2-butanone-4-phosphate synthase; CheA: histidine kinase; CheY: chemotaxis protein; CusR: cooper-transcriptional regulator; CusS: cooper-sensing histidine kinase; K1, E1: synthetic coiled-coils; P_*const*_: strong, constitutive promoter derived from *rsaA* gene encoding S-layer protein; P_*cusC*_: promoter derived *cusC* gene encoding efflux system protein and activated by phosphorylated CusR; PEM: proton exchange membrane; PmrA: iron-transcriptional regulator; PmrB: iron-sensing protein; P_*phyt*_: promoter derived from phytase-encoded operon and activated by phosphorylated UrpR; P_*pmrC*_: promoter obtained from *pmrC* gene encoding phosphoethanolamine transferase and activated by phosphorylated PmrA; P_*T7*_: T7 promoter; P_*urcB*_: promoter derived from *urcB* gene and activated by phosphorylated UzcR; UrpR, UzcR: uranium-transcriptional regulator; UrpS, UzcS: uranium-sensing histidine kinase.

In a study, electrical signal (voltage) was used as an output signal instead of color or fluorescence signals (Zhou et al., 2021). In *E. coli*, the *ribB* gene (3,4-dihydroxy-2-butanone-4-phosphate synthase) was transcribed for the production of riboflavin via the P_*cusC*_ promoter in response to Cu (Figure 4A). In addition, a porin gene (*oprF*) was constitutively expressed via T7 promoter, which exports riboflavin to the outer space. The escalation of extracellular riboflavin led to the increase in voltage by the electron transfer from riboflavin to anode. The electrical biosensor showed an LOD of 28.5 µM Cu^2+^, which offered a quick and affordable analytical method to biosensors for the *in situ* monitoring of Cu in water.

#### 3.2.2 Zn biosensor

It was discovered that the ZraSR TCRS responded to an extracellular Zn^2+^ level more specifically than other metal cations (i.e., Cd^2+^ and Pb^2+^) (Leonhartsberger et al., 2001; Appia-Ayme et al., 2012). The TCRS was employed in *E. coli* and the *gfp* gene was cloned downstream of the P_*zraP*_ promoter regulated by the ZraR regulator. The biosensor responded proportionally to Zn^2+^ and its LOD was as low as 1 µM (Ravikumar et al., 2011).

Another Zn^2+^-sensing system also utilized ZraSR TCRS via P_*zraP*_ promoter to regulate the *gfp* expression in response to Zn^2+^. A positive feedback loop was initiated by overexpressing the *zraR* gene encoding ZraR regulator downstream of *gfp* gene to enhance the sensitivity in LB media to 10 µM (Pham et al., 2013). However, when this system was integrated into the genome of *E. coli*, its sensitivity was reduced to 500 µM. Despite this reduction, this genome-integrated system still was valid as a biosensor for detecting Zn^2+^ in heavily contaminated areas with elevated concentrations of Zn^2+^. The study provided evidence for the practicability of biosensors utilizing TCRS in the detection of Zn^2+^ across a wide range of concentrations.

#### 3.2.3 Uranium (U) biosensor

Two TCRS for U sensing (UzcRS and UrpRS) have been discovered in uranium resistance bacterial *Caulobacter crescentus*, respectively (Park et al., 2019; Park and Taffet, 2019). For high specificity in U detection, the two TCRS systems (UzcRS and UrpRS) were employed and the two systems controlled the expression of two different fragments of whole GFP (GFP10-K1 and E1-GFP11), respectively (Figure 4B) (Cabantous et al., 2013; Park and Taffet, 2019). In the *C. crescentus*, the last fragment of GFP (GFP1-9) was constitutively produced. When the three different fragments were assembled, green fluorescence was emitted. Thus, the GFP fragments operated as an AND gate, and the gate was activated only when both the two TCRS systems detected U. The LOD of the U biosensor was 5 µM. In addition, the UrpRS system regulated the additional expression of the membrane protein UzcY that acts as the signal amplifier of the UzcRS system by improving the activity of UzcS to sense U. This regulation led to an increase in biosensor sensitivity, ultimately improving the sensitivity of the LOD to 1 µM.

#### 3.2.4 Cd biosensor

Chemotaxis has been harnessed to sense heavy metal ions. Unlike other biosensors, the output of chemotaxis-employed biosensor is the movement towards a target heavy metal. Bacterial cells are able to travel to find a favorable environment: they move towards attractants such as sugars and amino acids, while they move farther away from repellents such as alcohols and heavy metal ions (Co^2+^, Ni^2+^) (Eisenbach, 2011). The chemotaxis of bacteria is also regulated by TCRS that comprises CheA (histidine kinase), CheY (response regulator), and CheZ (phosphatase) (Bi and Sourjik, 2018). When there are no attractants, CheA phosphorylates CheY, which leads to cell tumbling in place by assembling with flagellar motor. When attractants are available, CheY is dephosphorylated by CheZ and the dissociation of CheY from the flagellar motor allows for moving forward (Bi and Sourjik, 2018).

*E. coli* harbors the Trg receptor that mediates chemotaxis to ribose via an interaction with the periplasmic ribose-binding protein. The ribose binding protein was engineered to sense Cd ions. Consequently, the engineered *E. coli* host with the Cd-sensing protein showed chemotactic migration towards Cd^2+^ (Figure 4C) (Li et al., 2022a). The engineered chemotaxis could foster intelligent bioremediation by moving microbial cells to a contaminated site.

#### 3.2.5 Lanthanide (Tb) biosensor

The PmrA/PmrB system, a TCRS for Fe^3+^-sensing in *Salmonella,* was engineered for the detection of lanthanide-ion (Tb^3+^) in *E. coli* (Liang et al., 2013). For sensing Tb^3+^, the Fe^3+^-binding loop in the histidine kinase of the TCRS was replaced with a Tb^3+^-binding peptide. The redesigned PmrA/PmrB TCRS system was capable of sensing Tb^3+^ at around 1.0 μM (Figure 4D). The repurposed sensing system was used to control the chemotaxis of *E. coli* towards Tb by regulating the expression of the CheZ protein. When CheZ was produced more due to high concentration of Tb, the engineered biosensor host could move closer/faster towards Tb than other cells producing less CheZ proteins (Bi and Sourjik, 2018).

## 4 Microbial remediation of heavy metals assisted by synthetic biology

In this section, microorganisms engineered for bioremediation are introduced by their remediation mechanisms. Of the three resistance mechanisms introduced in Section 2, sequestration is commonly utilized for bioremediation. Thus, constructed synthetic bacteria, to date, based on the heavy metal sequestration are introduced in this section.

### 4.1 Synthetic bioremediation bacteria based on extracellular sequestration

Certain bacterial species can trap heavy metals on their surface to prevent the penetration of heavy metals into the cells. The trapping is mediated via the interaction between functional groups attached on the cell membrane (amino, carboxyl, phosphate, and hydroxyl groups) and metal ions (El-Helow et al., 2000; Taniguchi et al., 2000). In addition, heavy metals can be also captured by extracellular polymeric substances secreted by bacteria, such as polysaccharides, mucopolysaccharides, and proteins (Zeng et al., 2020). However, the low inherent capture efficiency and instability of the extracellular polymeric substances prevent this approach from being broadly applied to the construction of synthetic bioremediation bacteria (Tang et al., 2021).

Recently, the advance in synthetic biology enabled us to develop metalloproteins and metal-binding peptides to exhibit improved heavy metal biosorption on the cell surface (Table 4) (Samuelson et al., 2002). The metalloproteins or metal-binding peptides were displayed on the cell membrane in the aid of membrane-anchoring proteins. In Gram-negative bacteria, outer membrane proteins (i.e., ice-nucleation protein (INP), Lpp-OmpA, OmpA, OmpC, and LamB), fimbriae/flagella, and autotransporter have been widely used as anchoring proteins. Metal-binding proteins were fused with the anchoring proteins at N- or C-terminus, or both for the membrane display (Samuelson et al., 2002; Wernerus and Stahl, 2004). In Gram-positive bacteria, staphylococcal protein A (SpA) is an effective anchoring protein to display metal-binding proteins (Wernerus et al., 2001). SpA comprises an N-terminal signal peptide (either four or five IgG-binding domains with a region rich in proline and glycine) and a C-terminal section (membrane-anchoring region) (Samuelson et al., 2002).

**TABLE 4:** Synthetic biology approaches applied to heavy metal bioremediation in bacteria.

Heavy metal	Engineered bacteria	Genetic elements for bioremediation	Bioremediation		Application site	References
			Initial metal concentration	Removal efficiency		
Pb	*E. coli* BL21	Three Pb-binding proteins (PbrR, PbrR691, and PbrD) were fused to the N-terminal domain of the ice-nucleation protein	200 μM	942.1, 754.3, and 864.8 µmol/gDCW for PbrR, PbrR691, and PbrD, respectively	Soil (*N. benthamiana* seeds and plant)	Jia et al. (2020a)
Pb	*E. coli* TOP10	P_T7_ promoter expressed the fusion protein containing Pb-binding domain (PbBD) obtained from the PbrR and C-terminus of the surface display protein Lpp-OmpA	50 μM	34.4 μmol/gDCW	N.A	Hui et al. (2018)
Hg	*E. coli* BL21	Hg-binding peptide (CL) was conjugated to the N-terminal domain of the ice-nucleation protein	75 μM	95% removal in solution 51.1% removal in fish intestine (*Carassius auratus*)	*Carassius auratus* intestine	Liu et al. (2019)
	*E. coli* MC4100 strain PQN4	P_*merR*_ promoter drove the expression of *yfp* gene for yellow fluorescent signal or synthetic *csgBACEFG* operon for producing Hg-sequestering curli	1,200 ppb	200 ng/mgDCW	N.A	Tay et al. (2017)
Pb/Cd	*E. coli* BL21	SynHMB was inserted into the surface-exposed region of OmpA’s first loop. Type VI secretory system gene cluster was co-expressed. Magnetic nanoparticles were grafted with polyethyleneimine and diethylenetriamine pentaacetic acid	50 mg/L	>90% removal	N.A	Zhu et al. (2020)
Cd	*E. coli* TOP10	P_*cad*_ promoter controlled the expression of fusion protein created by the integration of cadmium binding domain (CdBD) derived from *cadR* gene (*P. putida* 06909) with C-terminus of the surface display protein Lpp-OmpA	12.5 μM	1.86 μmol/gDCW	N.A	Guo et al. (2021b)
Cu	*E. coli* MC4100	The expression of fusion of CueR and Lpp-OmpA was controlled by a P_R_P_L_ promoter and a temperature-sensitive repressor (*cI* ^*ts857*^). P_*copA*_ promoter regulated by CueR controlled *egfp* gene expression	25 μM	Cu-binding capacity was maintained at around 91.5% after five cycles	Wastewater	Wang et al. (2019)
Cu	*E. coli* DH5α	U-shaped Cu-binding peptide was developed by screening synthetic degenerate DNA fragments. The peptide was then conjugated at the C-terminus of the maltose-binding protein	4 mM	N.A	N.A	Gahlot et al. (2020)
Cu	*E. coli* BL21	Green fluorescent protein-HG (Cu-binding peptide) fusion protein was integrated into the N-terminal domain of the ice-nucleation protein	50 μM	85.6% removal	Mining and industrial wastewater	Liu et al. (2022b)
Ni	*B. subtilis* PY79	P_*cotB*_ promoter expressed the fusion of CotB protein and His_18_ peptide	3 ppm	30.66 nmol/mg DCW	N.A	Hinc et al. (2010)
Ni/Cd	*B. subtilis* DB104	CotE protein was used as an anchoring protein for His_12_ peptide	N.A	82.4 nmol/mgDCW (Ni), 79.1 nmol/mg DCW (Cd)	N.A	Kim et al. (2019b)
As	*E. coli* BL21(DE3)	*B. subtilis*-derived ArsR was expressed in *E. coli*	50 μg/L of monomethyl arsenic acid and dimethylarsinic acid	82.4% monomethyl arsenic acid, 96.3% dimethylarsinic acid	N.A	Yang et al. (2013)
As	*E. coli* BLR(DE3)	Elastin-like polypeptide-fused ArsR was overexpressed by P_T7_ promoter	0.385 μM As^3+^	100%	Contaminated water	Kostal et al. (2004)
As/Cd	*E. coli* BL21(DE3)	Tandem oligomeric human metallothionein-1A fused with glutathione S-transferase was overexpressed by P_tac_	100 μM	6.36 mg/gDCW (Cd) 7.59 mg/gDCW (As)	N.A	Ma et al. (2011)
Cu/Cd/Zn	*E. coli* BL21(DE3)	Trimeric *Sinopotamon henanense*-derived metallothionein fused with a small ubiquitin-related modifier was overexpressed by P_T7_ promoter	300 μM	1.80 μmol/gDCW (Cu) 1.94 μmol/gDCW (Cd) 1.32 μmol/gDCW (Zn)	N.A	Ma et al. (2019)
Cu/Cd/Zn	*E. coli* BL21(DE3)	The mutant *Sinopotamon henanense*-derived metallothionein with three mutations (S37C, K49C, and K53C) was overexpressed by P_T7_ promoter	300 μM	1.81 μmol/gDCW (Cu) 2.05 μmol/gDCW (Cd) 1.44 μmol/gDCW (Zn)	N.A	Li et al. (2021)
Ni	*E. coli* JM109	*nixA* gene from *H. pylori* was overexpressed by P_T7_ promoter. Pea metallothionein fused with glutathione S-transferase was overexpressed by P_tac_	10 mg/L	83.33 mg/gDCW	N.A	Deng et al. (2013)
Hg	*R. palustris*	Hg-transport system (MerP-MerT) was co-overexpressed with pea metallothionein fused with glutathione S-transferase	90 mg/L	75 mg/gDCW	N.A	Deng and Jia (2011)
As	*E. coli* JM109	*Schizosaccharomyces pombe*-derived phytochelatin synthase was overexpressed along with feedback-insensitive ɣ-glutamylcysteine synthetase to concentrate phytochelatin pool. As-transporter (GlpF) was further expressed. As-efflux pump (ArsAB) was deleted	10 μM	16.8 μmol/gDCW	N.A	Singh et al. (2010)
Cd	*E. coli* JM109	Cd-transporter (MntA) was co-expressed with *Schizosaccharomyces pombe*-derived phytochelatin synthase	20 μM	31.6 μmol/gDCW	N.A	Kang et al. (2007)

N.A: not available.

#### 4.1.1 Pb bioremediation

To construct Pb-capturing *E. coli*, three Pb-binding proteins (PbrR, PbrR691, and PbrD) derived from *C. metallidurans* were fused at the N-terminal domain of INP to display on the surface of *E. coli* BL21 (Jia et al., 2020a). When the linker between INP and a Pb-binding protein and their expression levels were optimized, the three proteins (PbrR-INP, PbrR691-INP, and PbrD-INP) showed remarkably higher Pb-adsorption capacities of 942.1, 754.3, and 864.8 mol/g dry cell weight (DCW), respectively than reported so far. When the engineered *E. coli* was utilized to clear Pb in soil and its evaluation was carried out with seed germination and plant growth of *Nicotiana benthamiana*, the germination and the growth in the decontaminated soil were improved. Specifically, when the growth was measured as biomass after 50 days of cultivation, the biomasses of the plants grown in the decontaminated soil by PbrR-INP, PbrR691-INP, and PbrD-INP were 0.74 ± 0.21 g, 1.34 ± 0.27 g, 0.73 ± 0.05 g, respectively. The biomass of the plant grown in the contaminated soil as a control was 0.10 ± 0.02 g.

The integration of the Pb-binding domain (PbBD) from the PbrR protein with the Lpp-OmpA facilitated the extracellular accumulation of Pb in *E. coli* (Hui et al., 2018). The PbBD presentation on cell surface enhanced Pb^2+^ biosorption by 1.92-fold and achieved a capacity of 34.4 μmol/gDCW. The enhanced biosorption capability of PbBD compared with the whole protein (PbrR) resulted from the superior transport and localization of the short PbBD on the bacterial surface.

#### 4.1.2 Hg bioremediation

A synthetic Hg-binding peptide, that is rich in cysteine, has been conjugated to the N-terminal domain of INP and was expressed and displayed on the membrane of *E. coli* BL21 (Liu et al., 2019). With the synthetic Hg-binding peptide, the engineered *E. coli* could capture 95% of Hg^2+^ from the Hg-contaminated solution at 75 μM (Khati et al., 2012; Liu et al., 2019). As there is increasing concern about Hg pollution in the ocean and its toxicity to fish, the engineered *E. coli* w applied to fish to protect them from Hg. When the synthetic *E. coli* was fed to fish (*Carassius auratus*), it protected the gut microbial consortium in the fish intestine and prevented the host from Hg toxicity and cleared up to 51.1% of Hg in the intestine.

Tay et al. utilized the P_*merR*_ promoter derived from *S. flexneri* to drive the expression of *yfp* gene for yellow fluorescent signal or the synthetic *csgBACEFG* operon for producing Hg-sequestering curli in *E. coli* (Tay et al., 2017). The promoter is responsive to Hg, and curli are thin, aggregative, and extracellular fibers that adsorb Hg^2+^ extracellularly (Hidalgo et al., 2010). The engineered *E. coli* could capture Hg^2+^ from the environment at the removal efficiency of roughly 200 ng/mgDCW.

#### 4.1.3 Pb and Cd bioremediation

**FIGURE 5:**
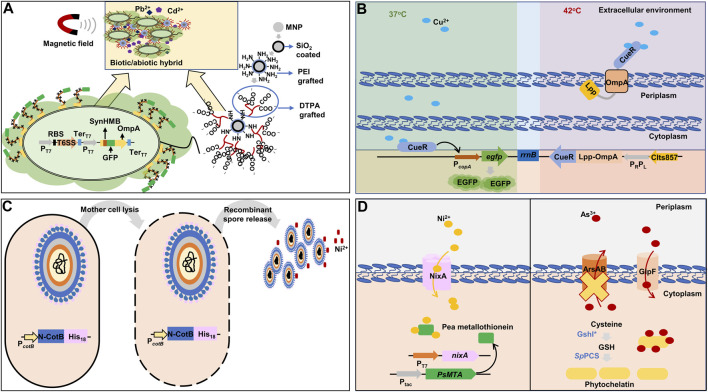
Representative synthetic biology approaches for heavy metals bioremediation in bacteria. **(A)** Combination of synthetic metal-binding peptide for improved extracellular sequestration of lead and cadmium, and chemically modified magnetic nanoparticles for easy recovery of bacterial cells after decontamination (Zhu et al., 2020). **(B)** A dual-functional synthetic bacteria for extracellular copper detection and biosorption regulated by thermal change (Wang et al., 2019). **(C)** Spore surface display with histidine residues to adsorbing nickel (Hinc et al., 2010). **(D)** Intracellular sequestration of nickel and arsenic by metallothionein and phytochelatin with the expression of respective heavy metal importers (Singh et al., 2010; Deng et al., 2013). The yellow cross denotes gene deletion. *nixA*: high-affinity nickel-transport protein; *PsMTA*: pea metallothionein; *rrnB*: terminator region derived from *rrnB* gene; ArsAB: arsenic exporter; CIts857: thermosensitive λ repressor; CueR: cooper-transcriptional regulator; DTPA: diethylenetriamine pentaacetic acid; EGFP: enhanced green fluorescent protein; Lpp: prolipoprotein; GlpF: arsenic transporter; GSH: glutathione; GshI*: feedback-insensitive ɣ-glutamylcysteine synthetase; His_18_: 18 histidine residues; MNP: magnetic nanoparticle; N-CotB: N-terminal domain of spore coat protein B; OmpA: outer membrane protein A; P_C_: constitutive promoter; P_*copA*_: promoter derived from *copA* gene encoding copper exporter and activated by copper-binding CueR; P_*cotB*_: promoter derived from *cotB* gene encoding spore coat protein B; PEI: polyethyleneimine; P_R_P_L_: tandem promoters of λ bacteriophage; P_T7_: T7 promoter; P_tac_: hybrids of the P_*lacUV5*_ and tryptophan (*trp*) operon promoter; RBS: ribosome-binding site; *Sp*PCS: *Schizosaccharomyces pombe*-derived phytochelatin synthase; SynHMB: synthetic heavy metals-binding peptide; T6SS: type VI secretory system gene cluster; Ter_T7_: T7 terminator.

In a study, a *de novo* synthetic heavy metals-binding peptide was implemented in *E. coli* BL21 and a chemically engineered magnetic nanoparticle was developed to efficiently capture Pb, Cd, and other heavy metals as well as to easily isolate the synthetic bioremediation bacteria (engineered *E. coli*) cells from decontaminated solution (Figure 5A) (Zhu et al., 2020). Firstly, a synthetic heavy metal-binding peptide, SynHMB, was inserted into the surface-exposed region within the first loop of OmpA. The type VI secretory system gene cluster of *P. putida* was additionally co-expressed to facilitate the display of the fusion protein (Bernal et al., 2017). Secondly, a magnetic nanoparticle was modified to contain polyethyleneimine and diethylenetriamine pentaacetic acid to interact with the engineered *E. coli* strain via the carboxyl groups in the nanoparticle and histidine residues of SynHMB expressed on the cell surface. The engineered *E. coli* strain efficiently removed over 90% of Pb and Cd from 50 mg/L of Cd^2+^- and 50 mg/L of Pb^2+^-containing solution. After decontamination, the cells were recovered using magnetic fields.

A system for both biosensing and extracellular sequestration of Cd was developed (Guo et al., 2021b). The system utilized the P_*cad*_ promoter and *cadR* gene derived from *P. putida* 06909 to produce an output signal (GFP) and a sequestration agent (a fusion protein of cadmium binding domain (CdBD) and Lpp-OmpA). The CdBD was derived from the CadR of *P. putida* 06909 and the chimeric sequestration agent (Lpp-OmpA-CdBD) captured Cd at cell surface. The engineered cell was capable of detecting Cd at a concentration as low as 0.1 µM, and their Cd-binding capacity was 1.86 μmol/gDCW.

#### 4.1.4 Cu bioremediation

A dual-functional *E. coli* that sensed and eliminated Cu ions was constructed, which switched its state from sensing to eliminating by temperature (Figure 5B) (Wang et al., 2019). Firstly, *E. coli* cell was designed to emit green fluorescence proportional to Cu ions in a sample. The *egfp* gene was placed under the P_*copA*_ promoter that was activated by the inherent Cu-binding transcription activator CueR. A fusion gene of the Cu-binding transcription activator CueR and Lpp-OmpA was constructed to capture Cu ions on the cell membrane. For the temperature-dependent transcription of the fusion gene, a P_R_P_L_ promoter and a temperature-sensitive repressor (*cI* ^*ts857*^) were used: the repressor maintained its repressor activity at 37°C, but lost its activity at 42°C and, thereby, the P_R_P_L_ promoter was activated. This dual-purpose *E. coli* cell responded specifically to Cu with a detection range of 0.01–25 μM at 37°C and exhibited Cu sequestration by CueR at 42°C. After 10 min of incubation at 42°C, the engineered strain could remove 91.5% of Cu ion in a water sample with 25 μM of Cu^2+^.

Lately, a putative U-shaped Cu-binding peptide was developed by screening synthetic degenerate DNA fragments, and the peptide was then conjugated at the C-terminus of the maltose-binding protein (MBP) (Gahlot et al., 2020). *E. coli* cells displaying the fusion of MBP and Cu-binding peptide demonstrated efficient Cu-binding and tolerance to up to 4 mM of Cu^2+^. This engineered cell possessed a Cu-binding capacity 32 times greater than that of the previously reported *E. coli* strain modified to sequester Cu^2+^ from Chinese wastewater (Wang et al., 2019).

A concurrent detection and biosorption bacterium has also been constructed (Liu et al., 2022b). A synthetic Cu-binding peptide, His-Gly-His-Gly-His-Gly-His (HG), was fused with GFP and the GFP-HG fusion protein was integrated into the N-terminal domain of INP for exterior display. The surface-engineered *E. coli* demonstrated Cu LOD down to 1 µM and adsorption up to 302.08 µmol/gDCW. In addition, since the Cu-binding peptide was fused with GFP, the binding between the peptide and Cu decreased the green fluorescence of GFP. Thus, the system could be also used as a biosensor for Cu.

#### 4.1.5 Ni bioremediation

*B. subtilis* spore surface display has been used to develop protein-coated spores to adsorb Ni ions (Lin et al., 2020). CotB, a protein on the surface of *Bacillus* spores, was modified to contain 18 histidine residues (His_18_) at its C-terminus since a poly-His tag is a well-known peptide to interact with Ni ions (Figure 5C) (Hinc et al., 2010). The recombinant spores showed better Ni elimination than wild-type spores and a higher capacity of approximately 30.66 nmol/mg of recombinant spore, compared with 24.79 nmol/mg of wild-type spore. Additionally, a shorter poly-His tag containing 12 His residues increased the capacity of the recombinant spores to 82.42 nmol/mg of recombinant spores (Kim et al., 2019b).

### 4.2 Synthetic bioremediation bacteria based on intracellular sequestration

Another sequestration mechanism harnessed to develop synthetic bacteria for heavy metals bioremediation is intracellular sequestration. Heavy metals are transported into the cytoplasm by appropriate importers and then sequestered by metal-binding proteins/peptides (Table 4) (Diep et al., 2018). The sequestered heavy metals are often chemically converted via enzymatic processes or precipitated.

#### 4.2.1 Intracellular sequestration by metalloregulatory proteins

Metalloregulatory proteins or heavy-metal responsive transcription factors induce the expression of proteins responsible for heavy-metal transporters, binding proteins, reducing proteins, etc. for heavy metals detoxification. Due to the excellent specificity and affinity to heavy metals, metalloregulatory proteins can be utilized to capture heavy metals inside cells.

When the ArsR proteins derived from *B. subtilis* were overexpressed in *E. coli*, the bacterial capacity to accumulate methylated As species was considerably increased (Yang et al., 2013). In another study, though ArsR proteins were overexpressed to increase accumulation capacity, the ArsR overproduction retarded cell growth significantly: the maximal OD decreased down to 25% of that of wild-type cells (Kostal et al., 2004). When elastin-like polypeptide was fused to ArsR as a solubilizing agent, cell growth was increased by two folds compared with the control while maintaining the same ability of ArsR to accumulate As^3+^. The synthetic bacteria overexpressing elastin-ArsR fusion protein resulted in 5- and 60-fold higher levels of As^5+^ and As^3+^ accumulation, respectively, compared with the control cells that did not express ArsR at all.

#### 4.2.2 Intracellular sequestration by metallothioneins

Heavy metal elimination in archaea, prokaryotes, and eukaryotes is complemented by metallothioneins that are abundant in nature. Metallothioneins have a strong affinity for heavy metals because of their cysteine-rich composition: the interaction between thiol groups in the cysteine residues and heavy metal ions. The aggregation of several heavy metals (Cu, Cd, and Mg) has been enhanced by overexpressing metallothioneins in microorganisms (Yang et al., 2015).

The intracellular overabundance of metallothioneins frequently experiences instability due to solubility issue, leading to a lowered heavy metal storage capability. An efficient solution to this issue is the co-expression of metallothioneins with a soluble fusion tag such as glutathione S-transferase (GST) and MBP. When human metallothionein (hMT-1A) was overexpressed in *E. coli* as a fusion protein with GST, the bacterium successfully accumulated Cd and As inside cells (Ma et al., 2011). Subsequently, oligomeric tandems of hMT-1A with various repeats were constructed to further improve Cd and As accumulation capacity. The maximum Cd and As bioaccumulation was achieved by the engineered *E. coli* expressing the GST-fused trimeric hMT-1A protein: 6.36 mg Cd^2+^/gDCW and 7.59 mg As^3+^/gDCW.

Ma et al. also utilized a similar approach for expressing *Sinopotamon henanense*-derived metallothionein (*Sh*MT) to sequester Cu, Cd and Zn in *E. coli* cells, but the difference was the use of small ubiquitin-related modifier (SUMO) as a solubility tag (Ma et al., 2019). Comparably, the SUMO-fused trimeric *Sh*MT demonstrated great bioaccumulation of Cu, Cd, and Zn: 5.8-fold, 3.1-fold, and 6.7-fold higher accumulation, respectively, than those of control cells expressing only SUMO.

Li et al. engineered the metallothionein (*Sh*MT) to improve its metal-binding ability. They conducted sequence-based alignment, structure-based molecular docking simulation, and site-directed mutagenesis to develop an efficient *Sh*MT mutant while not modifying the folding and molecular mass observed in the best oligomeric tandem design (Ma et al., 2019; Li et al., 2021). The *Sh*MT mutant with three mutations (S37C, K49C, and K53C), in which all the three residues were replaced with Cys, showed the maximum bioaccumulation of Cu, Cd, and Zn: 1.86 -fold, 1.71-fold, and 2.13-fold enhancement, respectively, than wild-type *Sh*MT.

The excellent capacity for heavy metal bioaccumulation of metallothioneins is countered by their complete absence of selectivity (Ma et al., 2011; Ma et al., 2019; Li et al., 2021). Hence, for the bioaccumulation of a specific heavy metal, instead of engineering metallothioneins, appropriate transports are often co-expressed to import specific heavy metals. For selective Ni-bioaccumulation, the Ni-affinity transmembrane protein originated from *H. pylori* (NixA) as well as GST-fused pea metallothionein were co-expressed (Figure 5D) (Deng et al., 2013). The synthetic bacteria expressing both NixA and GST-pea metallothionein showed a maximum Ni-uptake and accumulation capacity of 83.33 mg/gDCW.

When a metallothionein (GST-fused pea metallothionein) was co-expressed with the Hg-transport system (MerP-MerT) in a photosynthetic bacterium, *Rhodopseudomonas palustris*, the bacterium specifically accumulated Hg, but not other metal ions in solution (Deng and Jia, 2011).

#### 4.2.3 Intracellular sequestration by phytochelatins

Another form of cysteine-abundant peptide widely utilized to improve the capacity of bacteria to accumulate heavy metals is phytochelatins, which are found in plants or fungi (Schmoger et al., 2000). The common form of phytochelatins is (ɤ-Glu-Cys-)_n_-Gly (*n* = 1–11). Phytochelatins have a better adsorption affinity for heavy metals than metallothioneins since phytochelatins are longer and have a higher number of cysteine residues than metallothioneins, allowing them to form more stable complexes with heavy metals. Phytochelatin pools inside the cells can be boosted by increasing the level of phytochelatin precursors, cysteine, γ-glutamylcysteine, and GSH by overexpressing *cysE*, *gshI*, and *gshII*, respectively (Diep et al., 2018).

Singh et al. overexpressed *Schizosaccharomyces pombe*-derived phytochelatin synthase (*Sp*PCS) along with feedback-insensitive ɣ-glutamylcysteine synthetase (GshI*) in *E. coli*, which was shown to be the tailback of GSH formation (Singh et al., 2010). The synthetic bacterium showed a 30-fold increased phytochelatin level. In addition, for the specific bioaccumulation of As, As-transporter (GlpF) was additionally expressed while the As-efflux pump (ArsAB) was deleted from the genome of *E. coli* (Figure 5D). The resulting strain showed 80-fold increased As accumulation compared to the wild-type strain.

In another study, for the specific bioaccumulation of Cd, the Cd-transporter (MntA) was co-expressed with *Sb*PCS in *E. coli.* The engineered bacterium showed an increased Cd-specific storage capacity by 25-fold compared with the wild-type strain (Kang et al., 2007).

### 4.3 Difficulties in the application of synthetic biology in microbial bioremediation

The natural bacteria isolated from heavy metal-polluted sites have high potentials for bioremediation since they possess high tolerance and high removal efficiency. Though they are considered valuable platforms or chasses for synthetic bioremediation bacteria development, the current bacteria chasses used to implement genetic or regulatory elements for bioremediation are mostly laboratory model bacteria such as *E. coli*. The first hurdle is to isolate and cultivate natural bacteria (Bodor et al., 2020; Tran et al., 2021). Due to the complex requirements for the cultivation of isolated bacteria, it is difficult to obtain a pure bacterium and find the optimal growth conditions. As the isolated bacteria from contaminated areas are important sources of new elements for tolerance and detoxification, this difficulty hinders the advance of synthetic bioremediation bacteria construction. However, due to the advance in sequencing technologies and machine learning-based prediction models, new genes could be identified from a mixture of bacterial species without the need for bacteria isolation (Kobras et al., 2021; Konno and Iwasaki, 2023).

The second hurdle is the lack of available molecular toolkits for the non-model (isolated) bacterial organisms. This limitation hinders the construction of synthetic bacteria because natural bacteria that possess high bioremediation capability have far fewer molecular toolkits than common strains such as *E. coli*. Recent advances in synthetic biology toolkits that are universal, adaptable, and effective in various bacterial species could facilitate the modification of the non-model bacterial organisms, such as small regulatory RNAs (sRNAs) (Na et al., 2013), portable multiplex automated genome engineering (pORTMAGE) (Nyerges et al., 2016), clustered regularly interspaced short palindromic repeats (CRISPR)/Cas9 (Citorik et al., 2014), and so on.

## 5 Microbial detection and remediation: Limitations and current legal regulations

### 5.1 Limitations of microbial detection

Although whole-cell biosensors are promising tools for *in situ* heavy metal detection, they also have several limitations that may hinder their effectiveness. One limitation is the possibility of false-positive results due to the lack of specificity of biosensing systems (Guo et al., 2021b). Another limitation is the relatively high LOD in complicated environmental matrices, because interferents such as organic substances, minerals, and other metals could disrupt the detection ability of biosensors (Brányiková et al., 2020).

### 5.1 Limitations of microbial remediation

Bioremediation has advantages over chemical and physical remediation methods in terms of efficiency, sustainability, and ecofriendliness. However, it also has several drawbacks. Firstly, it is difficult to scale up from laboratory scale to industrial scale (Diep et al., 2018). While chemical and physical methods are predictable and thus easy to scale up, bioremediation employs living organisms that are very complex systems, and thus it is difficult to predict outcomes when the scale is changed.

Secondly, bioremediation mostly takes one or two strategies to remove heavy metals, mostly sequestration (Volesky and Holan, 1995). However, chemical and physical methods can utilize various strategies to remove heavy metals, including thermal treatment, adsorption, chlorination, chemical extraction, and electrokinetics (Selvi et al., 2019).

The last difficulty is the legal limitations. The wide use of GMMs in environments triggered a debate over the legality of employing GMMs in open environments (Singh et al., 2011). U.S. Environmental Protection Agency regulations require successful pilot tests before a technology may be used (Janssen and Stucki, 2020). Besides, the release of GMMs is restricted in most European nations unless authorization is acquired, and thus the use of GMMs in environments is not widely accepted (Wesseler et al., 2022). Therefore, the sluggish deployment of GMMs is ascribed to several safety concerns, legislative restrictions, and potential threats that the public may perceive, leading to strict control. It was predicted that advanced remediation procedures might be put off for up to 10 years due to these regulations (Janssen and Stucki, 2020).

During the heavy metal bioremediation, the introduction of GMMs to the fields can raise risks since GMMs do not stay in a controlled environment. The horizontal gene transfer might quickly occur between the GMMs and indigenous microorganisms (Sobecky and Coombs, 2009), and the delivery of recombinant genes may change the genetic makeup of native microorganisms (Kumar et al., 2020). The entry of recombinant genes into other species that can propagate the gene in the natural microbial populations may also stimulate bioremediation. However, the transfer of mobile elements like transposons or vectors containing antibiotic resistance genes triggers the emergence of naturally occurring super germs resistant to antibiotics (Wang and Zhang, 2019). Additionally, the alteration in microbial communities and metabolism may produce harmful substances. Thus, the U.S. National Institutes of Health (NIH) guideline suggested that recombinant *E. coli* should only escape at a maximum rate of 10^−8^ cells (NIH, 2019).

### 5.2 Current technical attempts to resolve the biosafety issues

There is an urgent demand for additional technologies to ensure the safe use of GMMs. Introducing genetic safeguards into engineered bacteria is examples of overcoming these restrictions (Moe-Behrens et al., 2013). For instance, toxin and antitoxin have been utilized to prevent horizontal gene transfer. The gene encoding toxin is harbored in a plasmid while the gene encoding for antitoxin is harbored in the genome (Paul et al., 2005). When the plasmid is transferred to another bacterial species that do not harbor the antitoxin gene, the bacteria will be killed by the toxin. However, mutations within the toxin gene may allow the successful transfer of the plasmid. To avoid the mutational escape, two or three separate systems can be introduced in the plasmid and the genome of synthetic bacteria. An example of this approach is that two *doc* genes encoding toxic Doc proteins and one *ecoRI* gene encoding a restriction endonuclease were cloned in three different plasmids. This approach prevents the mutational escape and the escape rate was below 10^−9^ when tested with *E. coli* and *P. putida* (Xue et al., 2022a; Xue et al., 2022b).

Controlled replication of plasmids is another method for safeguarding recombinant genes (Li et al., 2023). RepL, a replication protein that primarily regulates plasmid copy number, was introduced to a plasmid as a plasmid amplifier. The presence of an inducer for RepL expression increased the plasmid number, while the plasmid number was limited without an inducer. Furthermore, the conditional replication origin ColE2-P9 was introduced into the plasmid to control plasmid replication. The ColE2-P9 requires the initiator protein Rep that was introduced into the host’s chromosome (Yagura et al., 2006). Thus, when the plasmid was transferred to other bacteria, its replication was not initiated and its copy number was limited.

There is another strategy to control the growth of synthetic bacteria (Ronchel and Ramos, 2001). The *asd* gene engineered to be activated by XylS, which is essential for cell wall synthesis, was introduced into the genome of *P. putida* while the inherent native *asd* gene was disrupted. Another gene, a toxin *gef* gene that was engineered to be negatively regulated by XylS, was also introduced into the genome. When 3-methyl benzoate is not supplied to the host, which is an activator of XylS, the synthetic bacteria do not generate cell wall. Furthermore, the synthetic bacteria start produce a toxin protein from the *gef* gene and consequently dies because the cell does not have the antitoxin gene.

## 6 Conclusion and future perspectives

Heavy metal pollution is now becoming a global problem due to the numerous threats to human health and environment. Physical and chemical remediation have been widely applied to the elimination of hazardous heavy metals at an industrial scale. However, due to the concerns about sustainability, environmental friendliness, high cost, and the slow process, microbial remediation has gained attention as an alternative and innovative approach remediating heavy metals.

Non-model bacterial species predominate in nature and certain bacterial species may exhibit special metabolic features useful for bioremediation. Recent advances in synthetic biology could provide new genetic toolkits to upgrade the bioremediation capability of the microorganisms not only for laboratory bacterial species but also for non-model (isolated) microorganisms. However, operational reclamation of heavy metal-contaminated areas cannot be accomplished using a particular methodology. Physical, chemical, and biological methods must be combined to be entirely successful and comprehensive remediation, and this should be inspired the convergence of various fields.

Microorganisms can be an integrated platform or chassis of a heavy metal detection module and a remediation module, and the two modules can be linked to work in an orchestrated manner. Thus, they are highly effective in bioremediation compared with conventional methods in which detection and remediation are separated.

Future advance in engineering technologies with synthetic biology principles would pave the way for enhanced remediation platforms to safeguard human health and environments.
